# Expression and clinical significance of pyruvate kinase M2 in breast cancer: A protocol for meta-analysis and bioinformatics validation analysis

**DOI:** 10.1097/MD.0000000000025545

**Published:** 2021-05-07

**Authors:** Huayan Li, Min Yan, Xiaoyong Wu, Yanliang Wang, Lin Huang

**Affiliations:** Department of General Surgery, Hainan Western Central Hospital, Danzhou 571799, Hainan Province, China.

**Keywords:** bioinformatics, breast cancer, meta-analysis, protocol, pyruvate kinase M2

## Abstract

**Background:**

Breast cancer is a common malignant tumor in women. In recent years, its incidence is increasing year by year, and its morbidity and mortality rank the first place among female malignant tumors. Some key enzymes and intermediates in glycolysis are closely related to tumor development. Pyruvate kinase M2 (PKM2) is an important rate-limiting enzyme in glycolysis pathway. Meanwhile, it is highly expressed in proliferative cells, especially in tumor cells, and plays an important role in the formation of Warburg effect and tumorigenesis. Previous studies have explored the effects of PKM2 expression on the prognosis and clinical significance of breast cancer patients, while the results are contradictory and uncertain. This study uses controversial data for meta-analysis to accurately evaluate the problem. We collected relevant Oncomine and The Cancer Genome Atlas (TCGA) data to further verify the results. Through bioinformatics analysis, the mechanism and related pathways of PKM2 in breast cancer are explored.

**Methods:**

We searched Wanfang, Chinese Biomedical Literature Database, Chinese National Knowledge Infrastructure, the Chongqing VIP Chinese Science and Technology Periodical Database, PubMed, Embase, and Web of Science databases from inception to March 2021. The language restrictions are Chinese and English. The published literatures on PKM2 expression and prognosis or clinicopathological characteristics of breast cancer patients were statistically analyzed. Combined hazard ratios (HRs), odds ratios (ORs), and 95% confidence intervals (95% CIs) were used to evaluate the effects of PKM2 on the prognosis and clinicopathological features of breast cancer. Stata 14.0 software was applied for meta-analysis. Oncomine and TCGA databases were used to meta-analyze the differences of PKM2 mRNA expression between breast cancer and normal breast tissues. The expression of PKM2 protein was verified by Human Protein Atlas (HPA) database. The relationship between the gene and the survival of breast cancer patients was analyzed by Gene Expression Profiling Interactive Analysis (GEPIA). The relationship between PKM2 gene and clinicopathological characteristics was analyzed by using LinkedOmics, and the Kyoto Encyclopedia of Genes and Genomes (KEGG) enrichment pathway analysis was performed by using Metascape. Protein–protein interaction (PPI) network was constructed by String website.

**Results:**

The results of this meta-analysis will be submitted to a peer-reviewed journal for publication.

**Conclusion:**

This study provides high-quality medical evidence for the correlation between the expression of PKM2 and the prognosis and clinicopathological features of breast cancer. Through bioinformatics analysis, this study further deepens the understanding of the mechanism and related pathways of PKM2 in breast cancer.

**Ethics and dissemination:**

The private information from individuals will not be published. This systematic review also should not damage participants’ rights. Ethical approval is not available. The results may be published in a peer-reviewed journal or disseminated in relevant conferences.

**OSF REGISTRATION NUMBER:**

DOI 10.17605/OSF.IO/W52HB.

## 1 Introduction

The World Health Organization's International Agency for Research on Cancer has released the latest global cancer burden data for 2020.^[1]^ There are as many as 2.26 million new cases of breast cancer worldwide, with more than 2.2 million cases of lung cancer. Breast cancer has surpassed lung cancer as the largest cancer in the world. One of the fundamental reasons for the increase in the incidence of breast cancer is the changing risk factors of breast cancer, such as delayed childbearing and the reduction of birth, which is most evident in countries undergoing social and economic transition. Overweight, obesity, and lack of exercise are also responsible for the increase in the incidence of breast cancer worldwide. With the development of molecular diagnosis technology, early detection, diagnosis, and treatment have become key measures to reduce the mortality of breast cancer and prolong the survival time of patients.

The main manifestation of metabolic reprogramming of malignant tumor is aerobic glycolysis, and takes glycolysis as the primary productivity pathway even under the condition of sufficient oxygen, which plays an important role in the rapid growth of tumor cells.^[2–4]^ Pyruvate kinase M2 (PKM2) is a rate-limiting enzyme that is significant in the late stage of glycolysis.^[5–7]^ It can catalyze phosphoenolpyruvate to produce pyruvate and release energy, which is crucial in regulating cell metabolic activity and tumor growth.^[8,9]^ Clinical studies have revealed that the poor prognosis of many types of cancer is closely related to the overexpression of PKM2 that is considered as a potential diagnostic marker of malignant tumors such as lung, gastrointestinal tract, ovary, bile duct, and so on.^[7,10–12]^

Many studies have proved that the high expression of PKM2 is closely associated with the survival of breast cancer patients, but the results are uncertain.^[13–18]^ In order to explore the expression and clinical prognostic significance of PKM2 in breast cancer, we conducted a meta-analysis. Meanwhile, bioinformatics methods were adopted to analyze the expression of PKM2 in breast cancer and its potential biological process through public databases, including Gene Expression Omnibus (GEO), The Cancer Genome Atlas (TCGA), Human Protein Atlas (HPA), etc, so as to provide new ideas for further exploration of the molecular mechanism and therapeutic targets of breast cancer.

## 2 Methods

### 2.1 Study registration

The protocol of the systematic review has been registered on Open Science Framework. The registration number is DOI 10.17605/OSF.IO/W52HB. This meta-analysis protocol is based on the Preferred Reporting Items for Systematic Reviews and Meta-analysis Protocols (PRISMA-P) Statement Guidelines.^[19]^

### 2.2 Data sources and search strategy

Two independent reviewers searched several databases, including Wanfang, Chinese Biomedical Literature Database, Chinese National Knowledge Infrastructure, the Chongqing VIP Chinese Science and Technology Periodical Database, PubMed, Embase, and Web of Science, for studies on PKM2 and breast cancer. The publication date used to search the literature was from inception to March 2021. The search strategy for PubMed is illustrated in Table 1. The retrieval strategies of other electronic databases are carried out in accordance with PubMed.

**Table 1:** Search strategy in PubMed database.

Number	Search terms
#1	Breast Neoplasms[MeSH]
#2	Breast Cancer[Title/Abstract]
#3	Breast Tumors[Title/Abstract]
#4	Cancer of Breast[Title/Abstract]
#5	Cancer of the Breast[Title/Abstract]
#6	Human Mammary Carcinoma[Title/Abstract]
#7	Mammary Carcinoma, Human[Title/Abstract]
#8	Mammary Neoplasm, Human[Title/Abstract]
#9	Mammary Neoplasms, Human[Title/Abstract]
#10	Neoplasms, Breast[Title/Abstract]
#11	Tumors, Breast[Title/Abstract]
#12	Breast Neoplasm[Title/Abstract]
#13	Breast Tumor[Title/Abstract]
#14	Cancer, Breast[Title/Abstract]
#15	Carcinoma, Human Mammary[Title/Abstract]
#16	Carcinomas, Human Mammary[Title/Abstract]
#17	Human Mammary Carcinomas[Title/Abstract]
#18	Human Mammary Neoplasm[Title/Abstract]
#19	Human Mammary Neoplasms[Title/Abstract]
#20	Mammary Carcinomas, Human[Title/Abstract]
#21	Neoplasm, Breast[Title/Abstract]
#22	Neoplasm, Human Mammary[Title/Abstract]
#23	Neoplasms, Human Mammary[Title/Abstract]
#24	Tumor, Breast[Title/Abstract]
#25	or/1–24
#26	Pyruvate kinase M2[Title/Abstract]
#27	PKM2[Title/Abstract]
#28	or/26–27
#29	Prognos^∗^
#30	Survival
#31	or/29–30
#32	#25 and #28 and #31

### 2.3 Inclusion criteria for study selection

The inclusion criteria for studies are as follows:

1) All patients were diagnosed with breast cancer by pathology;
2) Specimens were derived from tumor tissues;
3) Studies evaluated the relationship between high PKM2 expression and survival or clinicopathological features in patients suffering from breast cancer;
4) Published languages were limited to Chinese and English;
5) The expression level of PKM2 in each study was divided into 2 levels based on cut-off value: high and low.

The literature exclusion criteria were:

1) Case reports, reviews, conference abstracts, and duplicate publications;
2) Animal tests.

### 2.4 Data collection and analysis

#### 2.4.1 Selection of studies

**Figure 1:**
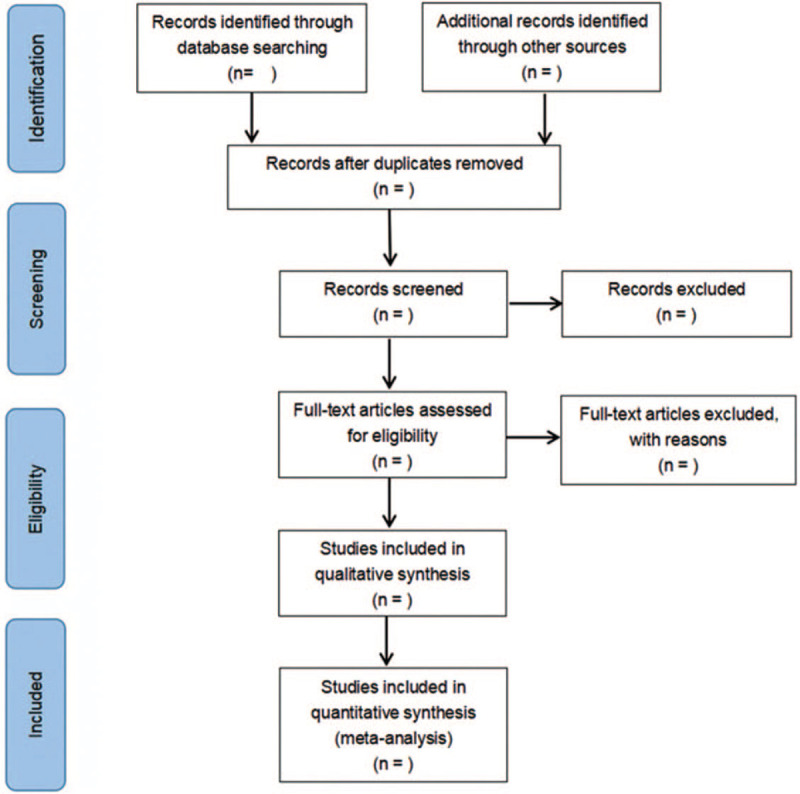
Flow diagram of study selection process.

The retrieved documents were imported into EndNoteX8 document management software. By reading the title and abstract, 2 evaluators screened the literature on the basis of the pre-established inclusion and exclusion criteria, then reviewed the full text of the literature that may meet the inclusion criteria, and finally determined the inclusion of the literature. If there is any objection, it will be resolved by a third independent evaluator. The literature screening process is displayed in Figure 1.

#### 2.4.2 Data extraction and management

Pre-made tables were used to extract data from the included literatures. The extracted contents mainly included:

1) basic information: first author, country, language, and PKM2 positive rate;
2) relevant clinical medical record data: sample size and age of each study;
3) pathological characteristics: tumor size, differentiation, lymph node metastasis (LNM), TNM stage, vascular invasion, and recurrence;
4) survival information: hazard ratio (HR) of overall survival (OS) and disease-free survival (DFS), corresponding to the 95% confidence interval (CI); Cox Multivariate Regression Analysis was preferred for survival data types; Kaplan–Meier survival curve obtained HR and 95% CIs by Engauge Digitizer version 4.1 (http://digitizer.sourceforge.net/).

### 2.5 Assessment of quality in included studies

Literature quality was evaluated based on the Newcastle-Ottawa Quality Assessment Scale (NOS).^[20]^ The perfect score is 9 points, and NOS score ≥ 6 is classified as a high-quality study.^[21]^

### 2.6 Measures of prognosis

OS and DFS will be taken as prognostic outcomes. The results will be expressed as HRs, with 95% CIs.

### 2.7 Management of missing data

If there is insufficient or missing data in the literature, we will contact the author via email. If the data is not available, we will only analyze the current available data and discuss the potential impacts.

### 2.8 Statistical analysis

STATA 14.0 (STATA Corporation, College Station, TX) was used for this meta-analysis, and HR and its 95% CIs were used to evaluate the relationship between PKM2 expression and clinical prognosis in patients with breast cancer. Pooled odds ratio (OR) and corresponding 95% CI were used for clinicopathological parameters. The chi-squared test and *I*^2^ values were carried out to assess the heterogeneity among the pooled analysis. When *P* > .1 and *I*^2^ < 50%, the fixed-effects model was adopted. By contrast, the random-effects model was adopted when *P* < .1 and *I*^2^ > 50%.

### 2.9 Additional analysis

#### 2.9.1 Subgroup analysis

We will conduct a subgroup analysis based on the detection method of PKM2 expression, race, publication year, sample size, and the sources of survival data.

#### 2.9.2 Sensitivity analysis

The sensitivity analysis of each index was performed through elimination method to check the stability of the results.

#### 2.9.3 Reporting bias

Publication bias was evaluated using Begg tests and defined significantly at a *P* < .05.^[22,23]^

### 2.10 Bioinformatics analysis

#### 2.10.1 Extraction of genetic data from Oncomine and TCGA databases

Oncomine database (https://www.oncomine.org/resource/login.html) is a bioinformatics analysis platform based on GEO (https://www.ncbi.nlm.nih.gov/geo/) and TCGA database (https://www.cancer.gov/about-nci/organization/ccg/research/structural-genomics/tcga). In this study, we first retrieve PKM2-related data sets in Oncomine database. The limiting conditions are as follows: Gene: PKM2; Cancer Type: Breast cancer; Analysis Type: Cancer vs Normal Analysis; Data Type: All; Gene Summary: *P* < .05; Order By: Over-expression Fold CHANGE > 2 Top gene RANK = 10%; Order By: Over-expression. Extracting the information about the expression of PKM2 gene in the data set, meta-analyzing each gene chip, and selecting the box map to display the results. Subsequently, the Gene Expression Profiling Interactive Analysis (GEPIA) platform (http://gepia.cancer-pku.cn/) based on TCGA database (https://www.cancer.gov/about-nci/organization/ccg/research/structural-genomics/tcga) was used to verify the expression of PKM2 in breast cancer.^[24]^

#### 2.10.2 Analysis of PKM2 protein expression by HPA database

HHPA database (https://www.proteinatlas.org/) provides tissue and cellular distribution information of a variety of human proteins using immunoassay technology.^[25,26]^ In this study, PKM2 antibody was selected to analyze the results of immunohistochemical experiment on normal breast tissues and breast cancer tissues.

#### 2.10.3 GEPIA online survival analysis

Breast cancer data from GEPIA database (http://gepia.cancer-pku.cn/) focus on evaluating the effects of PKM2 expression on OS of breast cancer patients.^[24]^

#### 2.10.4 Clinicopathological features and KEGG analysis

The LinkedOmics analysis platform (http://www.linkedomics.org/login.php) contains multiple sets of data on 32 cancers in the TCGA database. Entering the LinkFinder module and setting the target tumor to “breast cancer” and the target gene to “PKM2.” Clinical dataset was selected to obtain clinical information of breast cancer patients, and RNAseq dataset was selected to obtain PKM2 coexpression genes. Positive correlation genes with *P* < .001 and Statistic > 0.4 were screened and introduced into Metascape tool for gene Kyoto Encyclopedia of Genes and Genomes (KEGG) pathway enrichment analysis.

#### 2.10.5 Protein–protein interaction

An online analysis platform, the String website (https://www.string-db.org/) dedicates to the study of known and predicts protein-to-protein interactions. In this study, the website was used to predict the 10 proteins that are closely related to PKM2, so as to build a visual protein–protein interaction (PPI) network.

### 2.11 Ethics and dissemination

The content of this article does not involve moral approval or ethical review and would be presented in print or at relevant conferences.

## 3 Discussion

Pyruvate kinase (PK) is an important regulatory protein involved in glucose catabolism. There are 4 different subtypes of PK in mammals to meet the specific energy needs of different tissues.^[27]^ As a splice variant of PKM1, PKM2 has been discovered in embryonic cells, adult stem cells, and various tumor cells.^[28]^ PKM2 is directly related to metabolic reprogramming and inflammation of cancer, and its high expression level has adverse effects on tumor growth and prognosis.^[29]^

Lin et al found that the inhibition of PKM2 mRNA expression can down-regulate vascular endothelial growth factor C mRNA and protein and the inhibit cell proliferation.^[14]^ Dong et al proposed that high expression of PKM2 protein predicted poor progression-free survival and overall survival.^[15]^ Zhu et al put forward that the expression level of PKM2 was significantly correlated with tumor size, TNM stage, and lymph node metastasis of breast cancer.^[18]^ Therefore, as the final rate-limiting enzyme in the process of glycolysis, PKM2 is very important for the occurrence and development of breast cancer. In this study, meta-analysis and a variety of bioinformatics databases were applied to further explore the biological role and molecular mechanism of PKM2, thus providing a theoretical basis for the diagnosis and prognosis of breast cancer.

## Author contributions

**Conceptualization:** Lin Huang, Huayan Li.

**Data curation:** Huayan Li and Xiaoyong Wu, Lin Huang, Min Yan.

**Formal analysis:** Min Yan.

**Funding acquisition:** Lin Huang.

**Methodology:** Huayan Li, Min Yan.

**Project administration:** Lin Huang.

**Resources:** Xiaoyong Wu.

**Software:** Xiaoyong Wu.

**Supervision:** Min Yan, Xiaoyong Wu, Lin Huang.

**Validation:** Yanliang Wang.

**Visualization:** Yanliang Wang.

**Writing – original draft:** Huayan Li, Lin Huang and Min Yan.

**Writing – review & editing:** Huayan Li, Lin Huang and Min Yan.
